# Deep Eutectic Solvents for Improving the Solubilization and Delivery of Dapsone

**DOI:** 10.3390/pharmaceutics14020333

**Published:** 2022-01-30

**Authors:** Sonia Trombino, Carlo Siciliano, Debora Procopio, Federica Curcio, Annarita S. Laganà, Maria Luisa Di Gioia, Roberta Cassano

**Affiliations:** Department of Pharmacy, Health and Nutritional Sciences, University of Calabria, 87036 Rende, Italy; sonia.trombino@unical.it (S.T.); carlo.siciliano@unical.it (C.S.); debora.procopio@unical.it (D.P.); federica.curcio@unical.it (F.C.); annaritalag@virgilio.it (A.S.L.)

**Keywords:** deep eutectic solvents, green solvents, dapsone, API, drug solubility, drug delivery

## Abstract

Owing to a growing awareness toward environmental impact, the use of safer and eco-friendly solvents like deep eutectic solvents (DESs), has recently undergone important growth in the pharmaceutical field, with regard to their application as non-aqueous liquid administration vehicles, since they do not carry the same risks of toxicity and handling as traditional organic solvents. Major attention has been given to the development of advantageous transdermal drug delivery systems, because of their ease of use and better acceptability. Here, we report the use of two different DESs, based on choline chloride, used as hydrogen bond acceptor (HBA), and ascorbic acid or propylene glycol, used as hydrogen bond donors (HBDs), able to enhance the solubility and the topical delivery of dapsone, representing a class IV drug. The interactions between the DESs’ components and the drug were studied by performing DSC, FT-IR, and NMR analysis of the eutectic systems and the pure drug, confirming the establishment of H-bonds between the drug and the DESs’ components. Diffusion and permeability studies, carried out in a Franz cell, showed an increase in permeability, highlighting the great potential of DESs as dissolution and permeation enhancers in the development of novel and more effective drug delivery systems in topical administration.

## 1. Introduction

With the increasing environmental and human health concerns, over the past two decades, Green Chemistry has become a new standard embraced for the development of less dangerous materials and chemicals that are safer for both the environment and consumers [1]. Solvents are one of the major classes of chemical products that are widely used in many contexts [2]. Although important to industry, there are obvious environmental hazards associated with solvents, being typically made of fossil resources and being flammable or toxic [3]. The greening of pharmaceutical industrial methodologies, whose aim is to search for new alternatives to replace polluting and hazardous solvents with safer ones, has received great interest in the scientific community [4]. An ideal solvent for pharmaceutical purposes should preserve the integrity of an active pharmaceutical ingredient (API) candidate and be compatible with the requirements for administration to human patients.

One of the main challenges in drug manufacturing and development is to increase the permeability and bioavailability by enhancement of the solubility of poorly water-soluble drugs [5,6]. The low water solubility of drugs largely limits their formulation and bioavailability and can also result in significant side effects. However, most of the traditional organic solvents are not acceptable for pharmaceutical applications and present considerable constraints for manufacturing. Furthermore, besides being toxic, many of them have an unpleasant odor or taste.

All these considerations require a search for new media, which would offer higher solubility of the drug, as well as ensure environmental and health safety. In this context, the use of deep eutectic solvents (DESs) as alternative vehicles for the preparation of pharmaceutical formulations has recently gained deep interest [7,8,9,10]. The discovery of the deep eutectic solvents was a breakthrough in the world of green chemistry. They can be considered as “green solvents”, since they fully obey the green chemistry metrics, avoiding the same risks of toxicity and handling as traditional organic solvents [11,12,13,14,15,16,17]. DESs are generally defined as mixtures of two or more compounds, which at a particular molar ratio present a strong depression on the melting point, lower than those of its individual counterparts, allowing to remain in liquid state even at room temperature.

Due to their high stabilization and solubilization power, DESs offer the ability to tune the solubility, permeation, and absorption of active pharmaceutical ingredients (API) by several folds when compared with water [18,19,20]. Therefore, they represent an unconventional method to improve the dissolution and in vivo absorption of API with low aqueous solubility and/or low permeability. Furthermore, DESs could limit phenomena like polymorphism or degradation that represent serious problems encountered during drug formulation [21].

Dapsone (DAP) is a promising therapeutic agent for a wide range of dermatological diseases, since it combines antimicrobial and anti-inflammatory activities [22]. Its first use dates back to 1940 for the treatment of leprosy. It was later tested for skin disorders such as acne or dermatitis herpetiformis [23].

Furthermore, its use as an antimalarial and antileishmanial drug has also been described [24]. Even today, after half a century, dapsone represents a drug widely used in the treatment of acne. Since its oral administration is limited by its low water solubility, low bioavailability, and by dose-dependent hematological side effects, the transdermal delivery is the main strategy currently used [25,26]. Currently, DAP is commercialized as a gel formulation (Aczone^®^) in different countries for the treatment of acne [27,28,29].

The high lipophilic character of DAP may be advantageous to efficiently allow the crossing of the lipid bilayer of cells in the gut. However, DAP is catalogued in the biopharmaceutics classification system (BCS) as a class IV drug because of its low permeability and low solubility in water [30]. This can be responsible for microbial resistance and can limit the amount of free drug available to be absorbed. Therefore, several approaches have been proposed to address and overcome these limitations [31,32].

To this aim, we investigated the potential of DESs in increasing the solubility and skin permeability profile of dapsone. Among the various DESs, choline chloride (CC)-based DESs are commonly used, since it is a B vitamin that plays important roles in cell metabolism. Therefore, the systems suitability for the following purpose was carried out by using two different cholinium DESs, comprising ascorbic acid and propylene glycol as hydrogen bond donor, respectively. Moreover, system compatibility with the model drug was given considerable attention using DSC, NMR, and FT-IR analysis. Finally, the envisaged systems were investigated to be embedded in in a topical dosage form (cream), aiming to provide a more effective delivery of DAP in a local delivery approach.

## 2. Materials and Methods

### 2.1. Materials

Dapsone or 4,4′-diamino-diphenyl sulphone (97% purity, ref A74807, Sigma-Aldrich, St. Louis, MO, USA), choline chloride (98% purity, ref C1879, Sigma-Aldrich, St. Louis, MO, USA), ascorbic acid (99% purity, ref A5960, Sigma-Aldrich, St. Louis, MO, USA), propylene glycol, and phosphate buffer solution (PBS) were used as received from Sigma Aldrich, without any further purification. In order to prepare water-free DESs, CC, PG, and AA were dried in a vacuum oven at 60 °C for 12 h before their use.

### 2.2. Preparation of DESs

Choline chloride (CC, mp 302 °C), ascorbic acid (AA, mp 194 °C), and propylene glycol (PG, mp −56 °C) were used for the DESs preparation. CC: AA eutectic system was prepared as shown by Silva et al. [18]. Briefly, in a round bottom flask, CC and AA were mixed at the molar ratio of 2:1 (mp 133.8 °C). The mixture was kept under constant stirring speed of 100 rpm by using a heating plate (Bibby Scientific Limited, Beacon Road, Stone, Staffordshire, UK), at 60 °C, until a clear eutectic liquid was formed. The same procedure was used to prepare CC: PG at the molar ratio of 1:3 as well as CC:DAP (1:1 and 1:2). After preparation, the DES were preserved in the desiccator because the compounds are highly hygroscopic.

### 2.3. Polarized Optical Microscopy (POM) Analysis

A small droplet of the prepared DESs (CC:AA, CC:PG, CC:DAP) was deposited on a microscopic slide for the observation at a magnification of 10×. Nikon ECLIPSE LV100N polarizing microscope (Nikon Corporation, Shinagawa Intercity Tower C, Tokyo, Japan) coupled with Nikon DS-Fi2 camera was used for recording the polarized light image. The absence of solid crystalline structure is evidenced by a polarized light image totally black [33].

### 2.4. DES Solubility Measurement

To determine the solubility of DAP in the prepared DESs, its excess amount was added to ~1 mL of the eutectic mixtures which were then kept under constant stirring for 24 h, in order to reach equilibrium conditions at room temperature. After the saturation, the samples were filtered with PTFE syringe filter with a 0.45 μm membrane, to separate the macroscopic solid from the liquid phase. Then, the liquid phase was diluted in methanol, and the amount of the dissolved drug was determined by using a validated UV method on a UV spectrophotometer (UV-530 JASCO, 28600 Mary’s Court, Easton). The absorbance of the solutions was measured at the API maximum absorption wavelength (294 nm). In order to determine the concentration of DAP, a calibration curve was used. To this aim, pre-weighed amounts of DAP were dissolved in methanol and their absorbance in the function of concentration was measured and expressed as mass of the drug dissolved in mL of the solvent (mg/mL).

### 2.5. Fourier Transform Infrared Spectroscopy (FT-IR) Analysis

The pure drug together with KBr power (FTIR grade) was first homogenized in an agate mortar. Instead, the DESs and the drug dissolved in DESs were analyzed by means of KBr sheet. Infrared (FTIR) spectra were recorded using a FTIR Perkin-Elmer 1720 spectrophotometer over the 4.000–400 cm^−1^ range at a rate of 0.5 cm/s. Fifty scans were recorded, averaged for each spectrum, and corrected against ambient air as background.

### 2.6. Differential Scanning Calorimetry (DSC) Analysis

**Figure 1:**
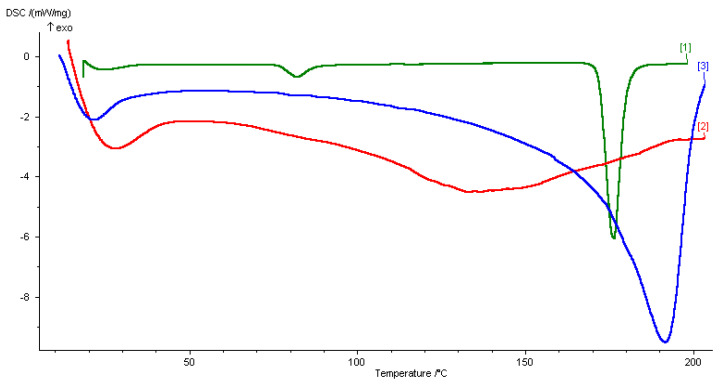
DSC thermograms of (**1**) dapsone, (**2**) drug-DES mixture, and (**3**) CC:PG DES.

The DSC experiments were performed for the different samples. Briefly, 5–9 mg of the DESs, solid drug, or the drug dissolved in DESs were placed in hermetic closed sample pans prior to thermal analysis using the DSC instrument (DSC 200 PC Netzsch, Wittelsbacherstraße 42, Selb, Germany). The thermograms were collected from 20 to 200 °C at a rate of 5 °C min^−1^, in order to detect all the transitions, melting points, and Tg of the substances. The DSC analysis of CC:AA DES (2:1) did not present the characteristic peaks of pure components, showing a strong melting point depression (Tf 133.8 °C), see Supporting Information. The thermogram obtained from the DSC analysis of the CC:PG DES (1:3) also did not present the characteristic peaks of the starting components, and showed an endothermic peak at a temperature of 21.8 °C (Figure 1).

### 2.7. NMR Analysis

NMR measurements were performed on a Bruker Advance 300 Ultra shielded spectrometer, operating at 300.132 and 75.08 MHz for ^1^H and ^13^C, respectively. The instrument was equipped with a 5 mm BB0 probe with *Z*-axis gradient coils, and an automatic temperature control unit. The samples were analyzed by using NMR 5 mm tubes (Wilmad). The temperature was set at 25 °C, with a variation of ±0.1 °C. Chemical shifts were reported in ppm and referred to the signal of dimethyl sulfoxide (central line of the quintet at 2.51 ppm for proton spectra, and central line of the septet at 40.00 ppm for carbon analysis). All spectra were obtained by applying pulse sequences from Bruker pulse program libraries. Acquisition and elaboration parameters were as elsewhere published [34].

*Choline chloride (CC)*. ^1^H NMR (δ): 5.70 (t, *J* = 6 Hz, 1H, OH), 3.75–3.87(m, 2H, CH_2_O), 3.35–3.47 (m, 2H, CH_2_N^+^), 3.17 (s, 9H, CH_3_); ^13^C NMR (δ): 67.33, 96.42, 53.51.

*4,4′-diamino-diphenyl sulphone (dapsone, DAP).* ^1^H NMR (δ): 7.48 (d, *J* = 9 Hz, 4H, ArH *o*-NH_2_), 6.61 (d, *J* = 9 Hz, 4H, ArH o-SO_2_), 5.98 (s, 4H, NH_2_); ^13^C NMR (δ): 153.22, 129.05, 128.66, 113.36.

*CC-PG DES.*^1^H NMR (δ): 5.55 (t, *J* = 3 Hz, 1H, OH ChCl), 4.57 (t, *J* = 6 Hz, 1H, CH_2_OH PG), 4.51 (d, *J* = 3 Hz, 1H, CHOH PG), 3.75–3.87 (m, 2H, CH_2_OH CC), 3.48–3.62 (m, 1H, CH PG), 3.40–3.47 (m, 2H, CH_2_N^+^ PG), 3.20–3.32 (m, 1H, CH_2_ PG), 3.15 (s, 9H, CH_3_ CC), 3.10–3.20 (m, 1H, CH_2_ PG), 0.99 (d, *J* = 6 H, 3H, CH_3_ PG); ^13^C NMR (δ): 67.78, 67.44, 55.60, 53.64, 20.48.

*DES-DAP.* ^1^H NMR (δ): 7.45 (d, *J* = 9 Hz, 4H, ArH o-NH_2_ DAP), 6.62 (d, *J* = 9 Hz, 4H, ArH *o*-SO_2_ DAP), 6.07 (bs, 4H, NH_2_ DAP), 5.53 (t, *J* = 3 Hz, 1H, CC), 4.65 (t, *J* = 6 Hz, 1H, CH_2_OH PG), 4.58 (d, *J* = 3 Hz, CHOH PG), 3.78–3.98 (m, 2H, CH_2_OH CC), 3.52–3.68 (m, 1H, CH PG), 3.39–3.48 (m, 2H, CH_2_N^+^ CC), 3.15 (s, 9H, CH_3_ CC), 3.09–3.35 (2 m, 4H, CH_2_ PG), 1.01 (d, *J* = 6 Hz, 3H, CH_3_ PG); ^13^C NMR (δ): 153.42, 129.12, 128.64, 113.52, 67.88, 67.54, 55.45, 53.77, 20.50.

### 2.8. In Vitro Skin Permeation

The permeation study (*n* = 3) was performed using Franz diffusion cells on skin removed from rabbit ears (2.9–3.1 kg New Zealand rabbits), previously sacrificed by a local slaughterhouse. Ears skins were shaved, separated, and immersed for 5 min in n-hexane to remove the debris adhered and then washed with distilled water.

Subcutaneous fat was removed using a bistoury, and the skin samples were washed with saline solution (NaCl 0.9%), and frozen at 20 °C. The obtained skin was allowed to equilibrate with dissolution medium (phosphate buffer pH 7.4) for 12 h before using it for permeation studies, and was mounted on Franz Diffusion cells having a surface area of 0.4614 cm2, and on receptor compartment having a capacity of 5.5 mL. Stratum corneum of the skin was exposed to ambient condition while dermal one was kept facing the receptor solution. The receptor compartment was filled with phosphate buffer as diffusion medium (34 ± 0.5 °C).

Dapsone-loaded DES were put on the stratum corneum and the donor compartment and at specific time intervals (15, 30, 60, 90, and 120 min), 1 mL of receiver solution was withdrawn from the receiver compartment and substituted with fresh buffer. The dapsone concentration in the receiver solution samples was analyzed by UV–Vis spectrophotometry. All experiments were performed in triplicate (*n* = 3).

### 2.9. UV-Vis Analysis

UV-Vis spectra were recorded with a Jasco V-530 UV/Vis spectrophotometer (JASCO, Halifax, NS, Canada). The absorbance of the sample was measured using quartz cells with a thickness of 1 cm and operating at a specific wavelength (293 nm). To evaluate the release profile, the content of five distinct samples taken at different time intervals (15, 30, 60, 90, 120 min) was analyzed. A phosphate buffer solution containing empty DES was used as a blank.

### 2.10. Statistical Analysis

Student *t*-test was used to evaluate the significance of data for release study. The test was performed by the ISTAT GraphPad software (San Diego, CA, USA). *p*-Values ≤ 0.05 were considered statistically significant.

## 3. Results and Discussion

### 3.1. Preparation of DESs

Over the years, different methods were proposed for preparing DESs, including vacuum, heating, and freeze-drying [35]. Out of them, heating was the major method used in this work, given its simplicity and common use.

Choline chloride was chosen as the hydrogen bond acceptor, while an organic acid, such as ascorbic acid, and a polyalcohol, like propylene glycol, were selected as hydrogen bonds donors. Choline chloride is a complex B vitamin that plays a vital role in cellular metabolism, commonly used as a food additive [36]. At the same time, ascorbic acid, known as ascorbate or vitamin C, can promote collagen biosynthesis, provide photoprotection, improve immunity, and reduce melanin production [37]. On the other hand, the use of DES based on choline chloride and ascorbic acid at the molar ratio of 2:1 has already been reported as an exciting possibility as solubilizing vehicle of active pharmaceutical ingredients (APIs) [18,38]. Moreover, as an alternative to the DES based on the use of organic acid as a donor, we decided to study the possibility of using a DES composed of choline chloride and a polyol as a donor [39,40]. Our choice fell on propylene glycol, often used as HBD, as it is a viscous liquid and a safer option to ethylene glycole, recognized in the pharmacopoeia as an excipient for the formulation of dermatological preparations. In addition, it is used as a non-toxic polyol in food-processing and for production of polymers [41]. Therefore, the DESs resulting by their combinations can be considered harmless, non-toxic, and advantageous for their applications such as drug delivery systems, given their natural and therapeutic properties and high biodegradability and biocompatibility. In order to confirm the formation of the eutectic mixtures, POM and DSC measurements were performed. The prepared DESs were well characterized by DSC, FT-IR, and NMR analysis. The results were in line with the literature [18,38,39,40] (Supporting Information).

Among the various possible types of DES, over the years, also THEDES, a new type of DES in which at least one of the components is an active ingredient, has been developed [42]. This class of DESs has a great potential as dissolution enhancer in the development of novel and more effective drug delivery system [43]. DAP is structurally characterized by the presence of groups that can act as donors and acceptors, resulting in a possible ideal component for the formation of THEDES. This strategy allows to overcome important challenges related to drugs and APIs, such as polymorphism, as well as increasing the solubility and bioavailability of the target API which leads to greater bioavailability. Based on these considerations, molar ratios 1:1 and 1:2 between choline chloride and DAP were tested, in order to obtain a stable and useful eutectic mixture for the intended purpose. However, none of the molar ratio tested satisfied the requirements (Supporting Information, Table S1). However, the two molar ratios considered do not lead to the formation of a DES useful for the intended purpose. The mixture of the two components in a 1:1 molar ratio lead to the formation of a viscous liquid that solidifies quickly at room temperature. At the same time, the mixing of components in a 1:2 ratio leads to a mixture that remained solid. These observations were corroborated by POM images where the presence of crystals were observed (Supporting Information, Table S1).

### 3.2. DESs Solubility Assay

DESs have been reported as an interesting alternative to improve the solubility and/or permeability of drugs and modulate their bioavailability [44]. Therefore, the potential of the prepared DESs in increasing the solubility of DAP was explored. The shake flask method represents a typical method for determining the solubility of pharmaceuticals in solvents [45]. In this process, an excess amount of the drug is added to a given volume of solvent, and the sample is placed under constant stirring at room temperature for a sufficient time to reach equilibrium. After equilibrium, the undissolved solid is separated from the solution, and the concentration of the dissolved drug is measured by an appropriate method.

DAP has a water solubility of 380 mg/L. The results showed that the solubility of DAP was largely increased in the DESs, compared to its solubility in water. In particular, the solubility of DAP in DES consisting of CC and AA amounts to ~150 mg/mL; a greater solubility of the same drug was found in the DES consisting of CC and PG, which turns out to be 500 mg/mL. Since propylene glycol is liquid at room temperature, it was interesting to verify whether this component could dissolve the drug. Therefore, the solubility of DAP in propylene alone was tested at the concentration of 500 mg/mL. Interestingly, it was observed that the addition of this quantity of dapsone led to the formation of a very turbid mixture, even after 48 h under constant stirring at rt. This result further confirmed the important solvent power of the DES considered, improving drug solubility. The successful dissolution of the drug in the DES systems was confirmed by POM images, since no crystal-like structures (black images) were observed when compared with drug in propylene glycol (Supporting Information, Figure S4).

CC:AA is characterized by high viscosity, given the extensive hydrogen bonds between the DES components. This can be a limiting factor affecting homogenization/solubilization [46]. The addition of water to the DES is generally used to reduce viscosity, modifying hydrogens bonds in DES, thereby facilitating mass transfer from the solute to the solution [47]. More importantly, the dissolving capacity of DES can be adjusted by varying the water content. Therefore, formulations containing 10% and 20% by volume of water in the DES-water mixture were tested to dissolve the model drug. Higher percentages showed to cause the DES solubilization by breaking up the hydrogen interactions among the components and promoting hydrogen interactions between the water molecules themselves, reducing the ability of the DES in enhancing dissolution. Although the addition of water greatly reduced viscosity, this did not significantly improve the solubility of the model drug in DES, reaching a maximum of 200 mg/mL in the DES with the 20% water content.

The increase in solubility of DAP in the DESs can be justified by the formation of hydrogen bonds between DAP and DES components. In order to detect intermolecular interactions between DAP and the DESs’ components, the DSC, FIT-IR, and NMR analysis were performed on the pure drug, the single DESs, and the drug-DES mixtures. In particular, the investigation mainly concerned the CC:PG DES, given its highest solvation power.

#### 3.2.1. DSC Analysis

DSC analysis of the pure DAP, the CC:PG DES, and the drug-DES mixture was adopted as an efficient technique to track thermal and crystallographic changes of the resulted mixtures. Generally, the thermogram of CC presents an endothermic peak at 81 °C which is ascribed to a crystallographic arrangement during its transition phase [48,49]. On the other side, the thermogram of PG is dominated by an endothermic peak at −59 °C, which corroborated previous results in the literature. Like all eutectic binary mixtures, the DSC thermogram of the CC:PG DES represents a complex formed by strong hydrogen bonding between the starting components. This was corroborated by the absence of the melting endotherms of propylene glycole and choline chloride peaks (Figure 1, thermogram 3). As a consequence, a strong depression on the melting points occurs.

The addition of the drug in the eutectic mixture did not significantly affect the main crystallization and melting peaks of the DES (Figure 1, thermogram 2). However, the melting endotherm of DAP at 176.7 °C (Figure 1, thermogram 1) no longer existed. The absence of the melting point of the drug in the thermogram of the drug-DES mixture highlighted the ability of the DES to maintain the drug in the soluble state up to temperature that is lower than its melting point.

#### 3.2.2. FT-IR Analysis

In order to gain deep insight into the intermolecular interactions between DAP and the CC:PG DES’s components, the infrared spectra of pure DAPs as well as DES and the drug-DES system have been performed and analyzed. Changes in the structure can be seen from the widening or merging of the peaks involving hydroxyl and amino groups, the shift of carbonyl peak of acids, or loss of the representative group peak of pure substances.

**Figure 2:**
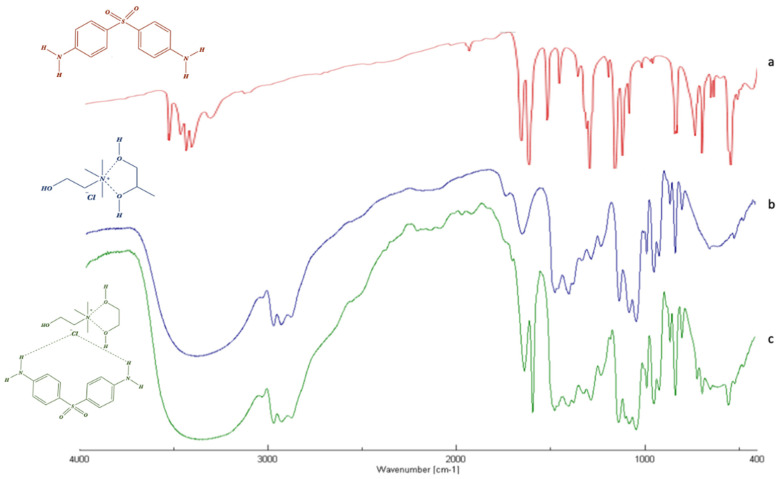
FT-IR of dapsone (red) (**a**), CC:PG DES (blue) (**b**) and drug-DES mixture (green) (**c**).

FT-IR spectrum of pure dapsone (Figure 2a) is characterized by a band at 3300 to 3400 cm^−1^, corresponding to the stretching of the amino group, and peaks between 1590 and 1550 cm^−1^, corresponding to the bending vibration of -NH_2_ groups. Other bands at 1143 and 1180 cm^−1^ are ascribed to the symmetric and asymmetric vibrations of the sulfone group (-SO_2_). Finally, the bending (out-of-plane) vibration of *p*-disubstituted aromatic ring can be found at 831 cm^−1^.

As shown in Figure 2c, the FTIR spectrum of the DAP-DES mixture was nothing more than a combination of the single DAP and DES spectra. In fact, all the previous bands are clearly observed in the spectrum of drug-DES system, with no radicle change related to the major vibrations of the single spectra. The most obvious differences were observed in the 3200–3600 cm^−1^ region, related to the elongation vibrations of hydroxyl groups (-OH) of the DES’s components and the stretching of the amino group (N-H) of the DAP, and in the 1140 and 1190 cm^−1^ region, ascribed to the symmetric and asymmetric vibrations of the sulfone group of DAP. The enlargement and shifting of such bands, respectively, are attributed to the formation of hydrogen bonds between the components. In all spectra analyzed, the appearance or disappearance of IR bands were not observed, suggesting that the drug preserved its chemical structure when it interacts with DES’s components.

#### 3.2.3. NMR Characterization

**Figure 3:**
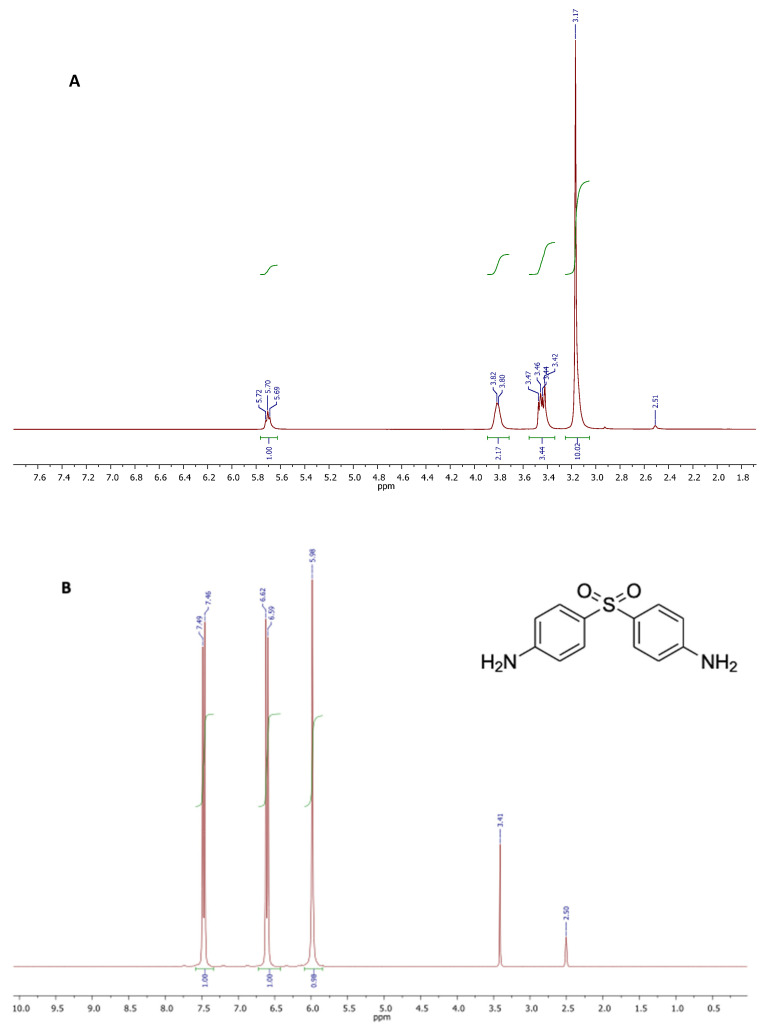
^1^H NMR spectra of choline chloride (CC) (**A**), and 4,4′-diamino-diphenyl sulphone (dapsone, DAP) (**B**).

The NMR spectra recorded on samples of choline chloride (CC) and DAP, dissolved with no pre-treatment in exadeuterated dimethylsulfoxide (DMSO-d_6_) as the solvent, showed the respective chemical structural features and the appropriate signal intensities (Figure 3, spectra A and B). DMSO-d_6_ was used as the solvent due to CC and DAP good solubility herein, and its weak effect on the interaction with the other components in DES. All proton and carbon resonances were attributed and confirmed comparing the experimental chemical shifts with those already reported in literature for the same compounds. Contrariwise, all proton and carbon signals of propylene glycol (PG) were attributed according to the literature [50].

The proton spectrum depicted in Figure 3A, showed the characteristic singlet generated by the three methyls of choline structure, centered at 3.17 ppm. Two multiplets at 3.38–3.49 ppm and 3.75–3.83 ppm were attributed to the protons of methylene groups bound to the quaternary nitrogen and the hydroxyl function, respectively. The triplet appearing at 5.70 ppm was generated by the OH proton resonance. The spectrum of DAP (Figure 3B) showed three signals: a singlet at 5.98 attributable to the four protons of the amino groups, and two doublets centered at 6.61 and 7.48 ppm attributable to the resonances of the aromatic protons in the ortho position with respect to the amino and sulphonic groups, respectively. The singlet at 3.41 was due to impurities.

**Figure 4:**
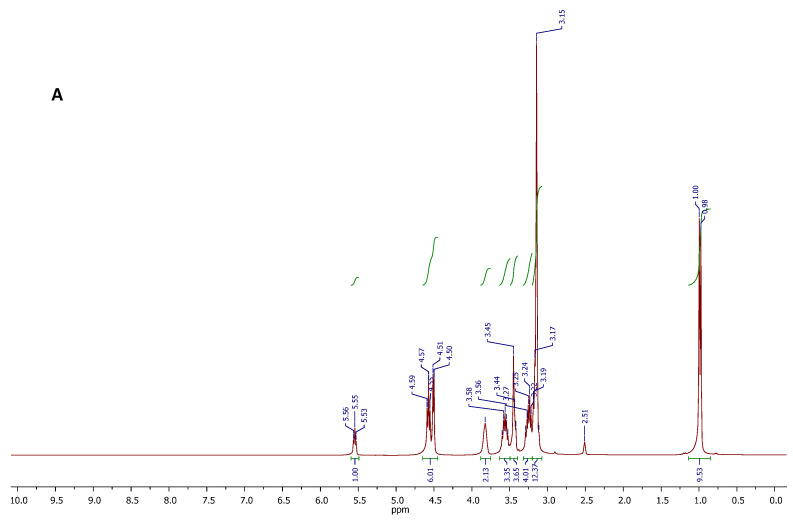

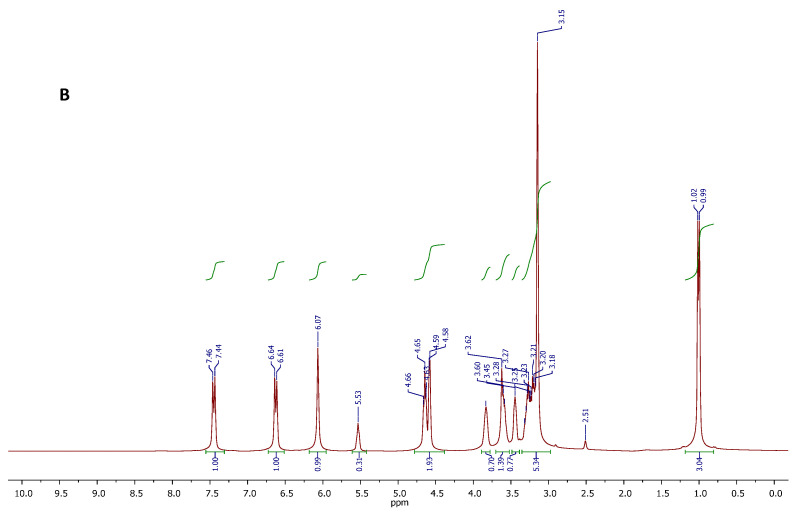
^1^H NMR spectra of the CC-PG DES (**A**), and DES-DAP ternary system (**B**).

The chemical structure and purity of the DES, and the DES-DAP system were likely confirmed by proton and carbon NMR spectroscopy. The proton spectra are displayed in Figure 4, while the respective carbon spectra are reported in the Supporting Information Section. In that case, the ^1^H NMR analysis was useful to confirm the existence of molecular interactions between the components of DES, on the basis of the chemical shift variations observed for some spin systems. With reference to the spectrum A in Figure 4, the DES showed all the expected proton resonance signals. In particular, signals appearing at 5.55 (triplet), 4.57 (triplet), and 4.51 (doublet) ppm, were due to the resonances of OH protons of CC and PG. The signal centered at 3.82 ppm, and the signal appearing between 3.40 and 3.50 ppm were respectively attributed to the CH_2_O and CH_2_N methylene protons of CC structure, while the well-defined signals centered at 3.24 and 3.56 ppm were generated by the CH_2_ protons of PG. The intense singlet at 3.15 ppm is referred to as the nine methyl protons of CC, and this signal overlaps the multiplet generated by the methine proton in the PG structure. Finally, the doublet at 0.99 ppm was attributed to the CH_3_ protons of PG. Integral values of all signals in the spectrum were found to be consistent with the molecular composition of the DES. No other signals were observed in the spectrum, indicating the DES purity. Comparing with the spectra of pure CC and PG, the chemical shift of OH of CC moved to upfield, and the OH protons of PG moved to downfield in the binary system CC-PG. It is likely that the intramolecular hydrogen bonds in CC were broken, and new intermolecular hydrogen bonds formed between CCC and PG.

After DAP was loaded into CC-PG DES, all the active hydrogen signals of each component in the formed ternary mixture were clearly distinguishable and attributable (Figure 4, spectrum B). The spectral region between 5.75 and 7.75 ppm showed all signals attributable to the resonance of the DAP protons. The signals of all C-H protons in the structures of CC and PG were found in the spectral region between 0.80 and 4.00 ppm. Furthermore, the ^1^H NMR spectrum of the DES-DAP system could infer the presence of interactions between DAP and the DES components. In fact, it is important to note the chemical shift variations of resonances attributed to NH and OH protons. In particular, the NH_2_ protons of DAP resonated at 6.07 ppm, showed a significant downfield shift with respect to the signal featured by the spectrum recorded for the sample of standard DAP (5.98 ppm), and suggesting the NH_2_ groups might be involved in the formation of H-bonding networks with the DES components.

### 3.3. Dapsone Release

**Figure 5:**
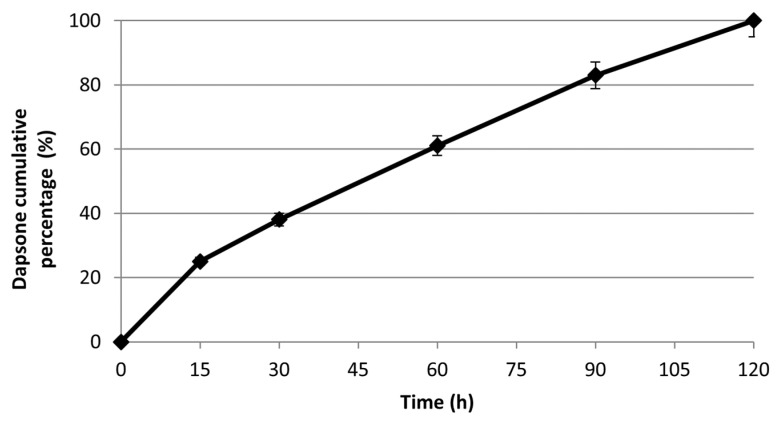
The in vitro cumulative release of Dapsone from the DES in pH 7.4 at 34 °C.

To evaluate the suitability of DES for dapsone release, in vitro studies were performed at different time intervals (15, 30, 60, 90, and 120 min) and at a pH 7.4 and 34 °C, with the aim to simulate physiological conditions. The drug release profile was determined by UV-Vis spectrometry and expressed as a cumulative percentage of the released drug as a function of time (=0.908, =293 nm). Data analysis showed a total and massive release of the drug in the first two hours while no release was observed in the following hours (Figure 5). This release profile can be considered very advantageous and useful for obtaining the desired therapeutic effect. In particular, the complete release of the loaded drug within 2 h results quite satisfactory.

## 4. Conclusions

In the present study we demonstrated that CC: PG DES can be a promising system to dissolve dapsone at high concentration, offering a solubility of 500 mg/mL as compared to its solubility in water (380 mg/L). This significant increase in solubility was attributed to the DES’s components ability in forming hydrogen bonds with the drug, as shown by FT-IR and ^1^H NMR analysis. DSC results further confirmed the success of the eutectic mixture in enhancing drug solubility, given the absence of the drug’s fusion endotherm. Finally, we found that in vitro drug release rendered it suitable for the transdermal delivery of dapsone in physiological pH conditions.

## Supplementary Materials

The following supporting information can be downloaded at: https://www.mdpi.com/article/10.3390/pharmaceutics14020333/s1, Table S1: Experimental POM images of the prepared DESs. Figure S1: FTIR spectra of choline chloride, ascorbic acid and CC:AA DES (2:1). Figure S2: FTI-R spectra of choline chloride, propylene glycol and CC:PG DES (1:3). Figure S3: ^13^C NMR spectra of choline chloride (a), dapsone (b), ChCl-PG DES (c) and DES-DAP ternary system (d). Figure S4: POM images of (DRUG + PG) and (DRUG + DESs) at rt. Figure S5: DSC thermograms of (1) CC:AA DES, (2) pure CC and (3) pure AA.
