# Supplementary material for: Deep Eutectic Solvents for Improving the Solubilization and Delivery of Dapsone
Source: Pharmaceutics. 2022 Jan 30;14(2):333. doi: 10.3390/pharmaceutics14020333 (PMC8875782; doi:10.3390/pharmaceutics14020333)
Supplement: Supplementary file 1 [file pharmaceutics-14-00333-s001.zip › pharmaceutics-1551325-supplementary.pdf]

pharmaceutics

MDPI

Supplementary Materials: Deep Eutectic Solvents for Improving the Solubilization and Delivery of Dapsone

Sonia Trombino, Carlo Siciliano, Debora Procopio, Federica Curcio, Annarita S. Laganà, Di Gioia Maria Luisa, and Cassano Roberta

Table S1. Experimental POM images of the prepared DESs.

DESs	HBA	HBD	HBA/HBD molar ratio	Aspect	POM images
**CC:AA**	CC	AA	2:1	Viscous yellow liquid	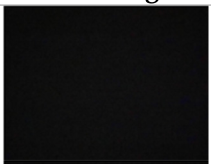
**CC:PG**	CC	PG	1:3	Transparent liquid	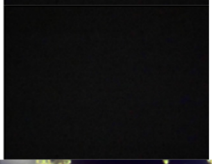
**CC:DAP**	CC	DAP	1:1	Viscous liquid that rapidly solified at rt	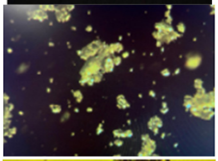
			1:2	Solid	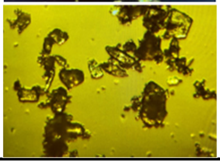

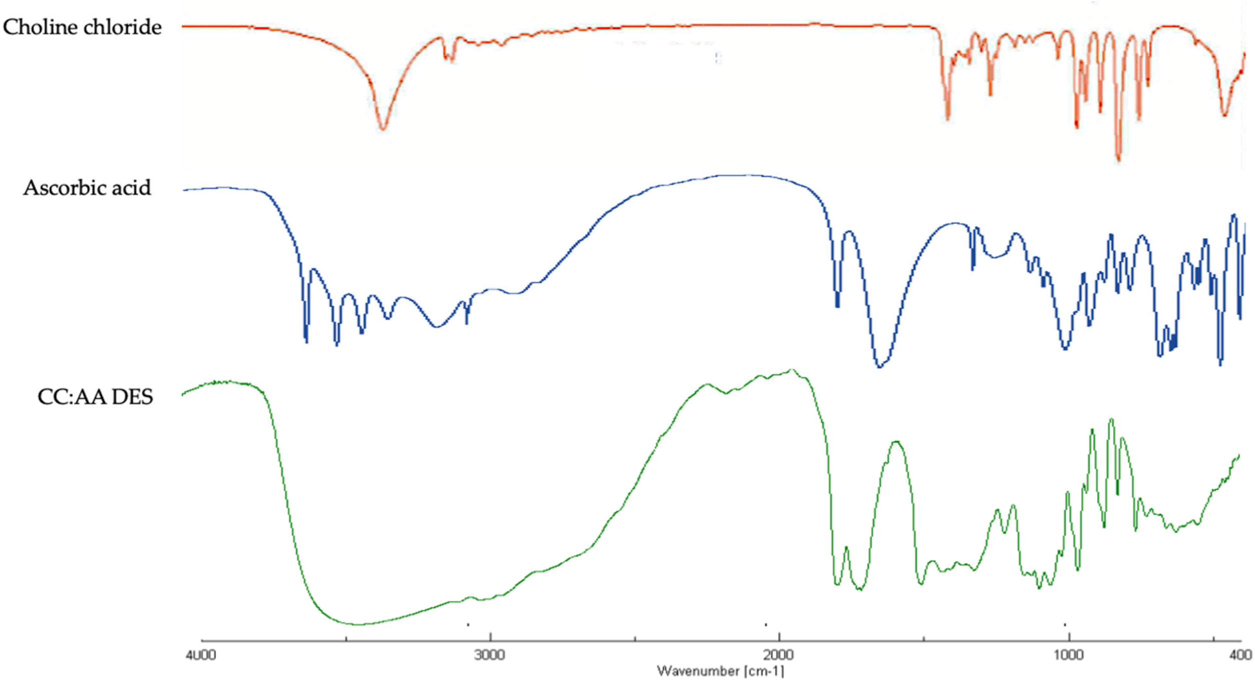

Figure S1. FTIR spectra of choline chloride, ascorbic acid and CC:AA DES (2:1).

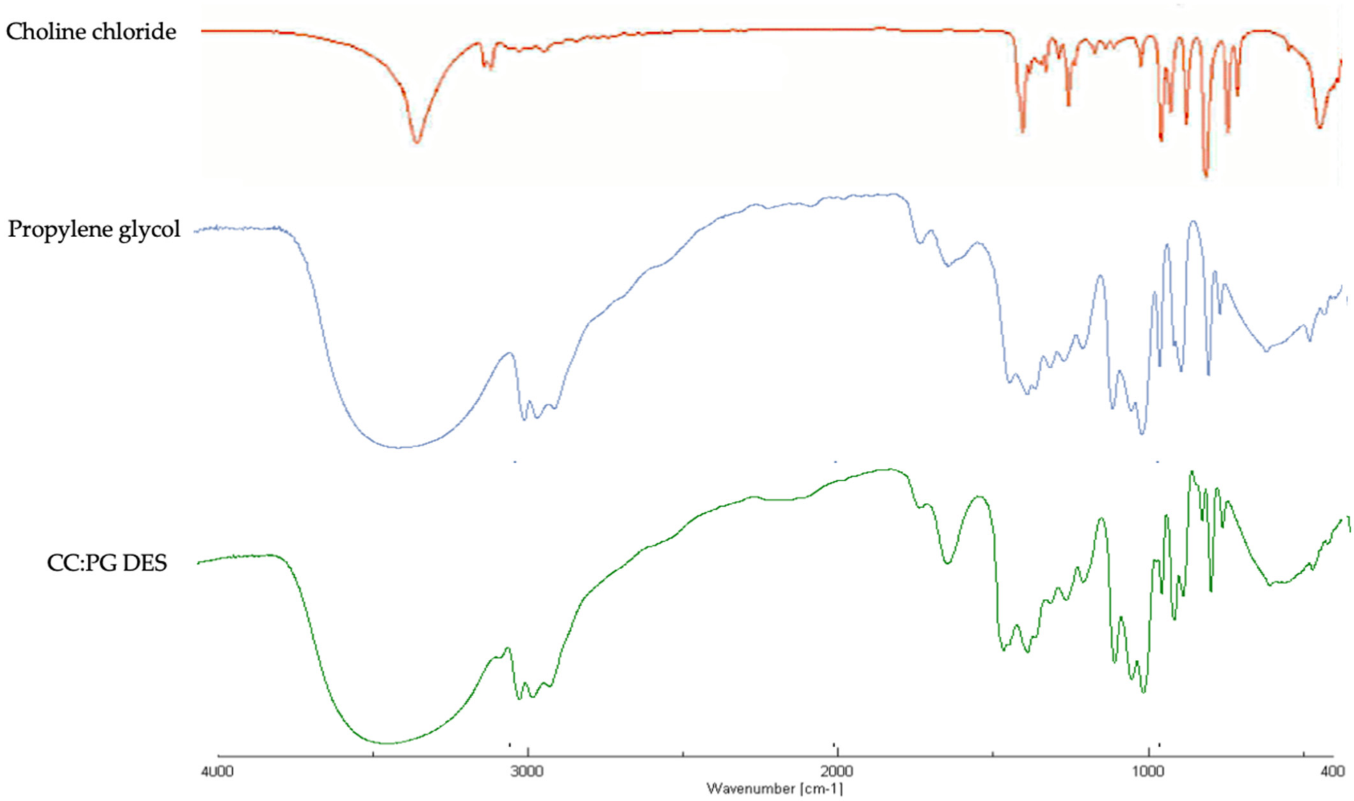

Figure S2. FTIR spectra of choline chloride, propylene glycol and CC:PG DES (1:3).

a

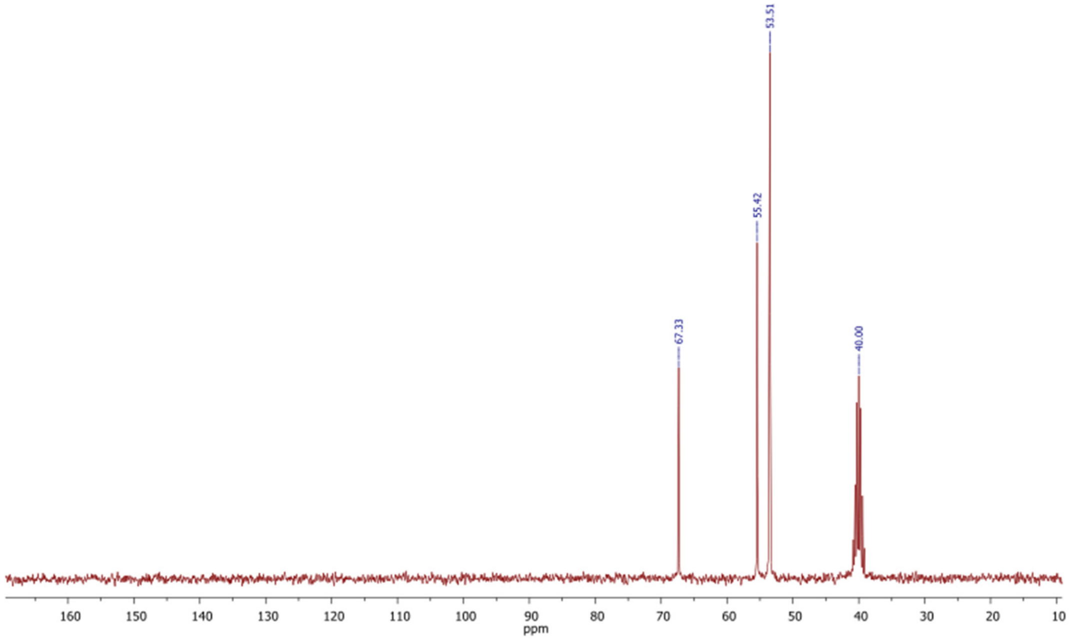

b

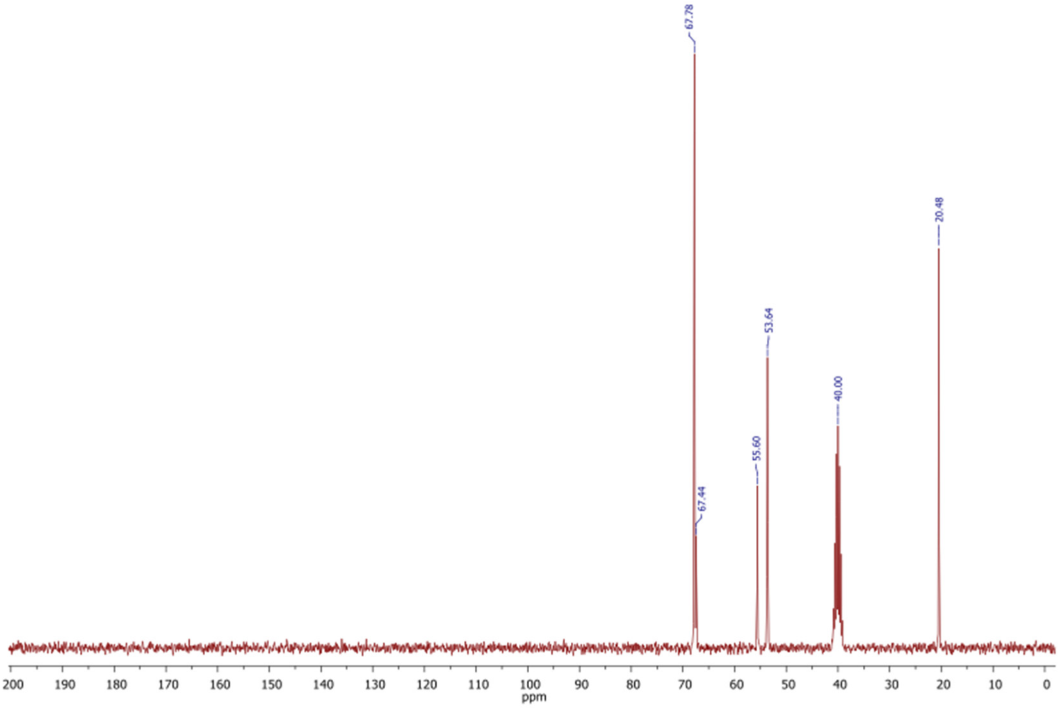
67.78
67.44
55.60
53.64
40.00
20.48
200 190 180 170 160 150 140 130 120 110 100 90 80 70 60 50 40 30 20 10 0
ppm

c

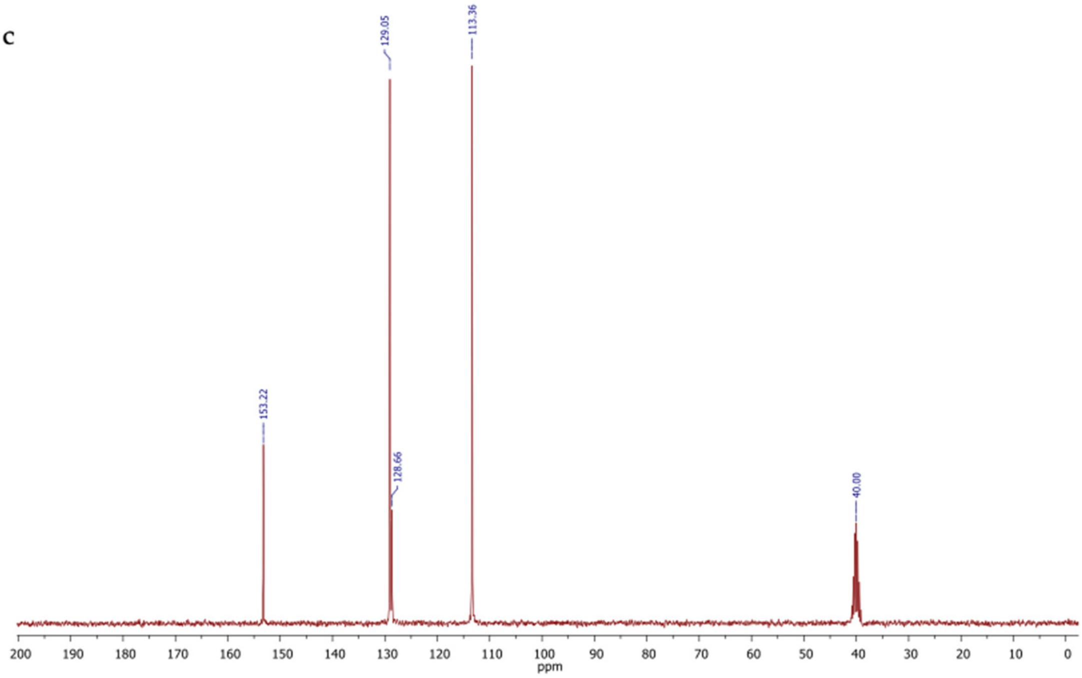
153.22
129.05
128.66
113.36
40.00
200 190 180 170 160 150 140 130 120 110 100 90 80 70 60 50 40 30 20 10 0
ppm

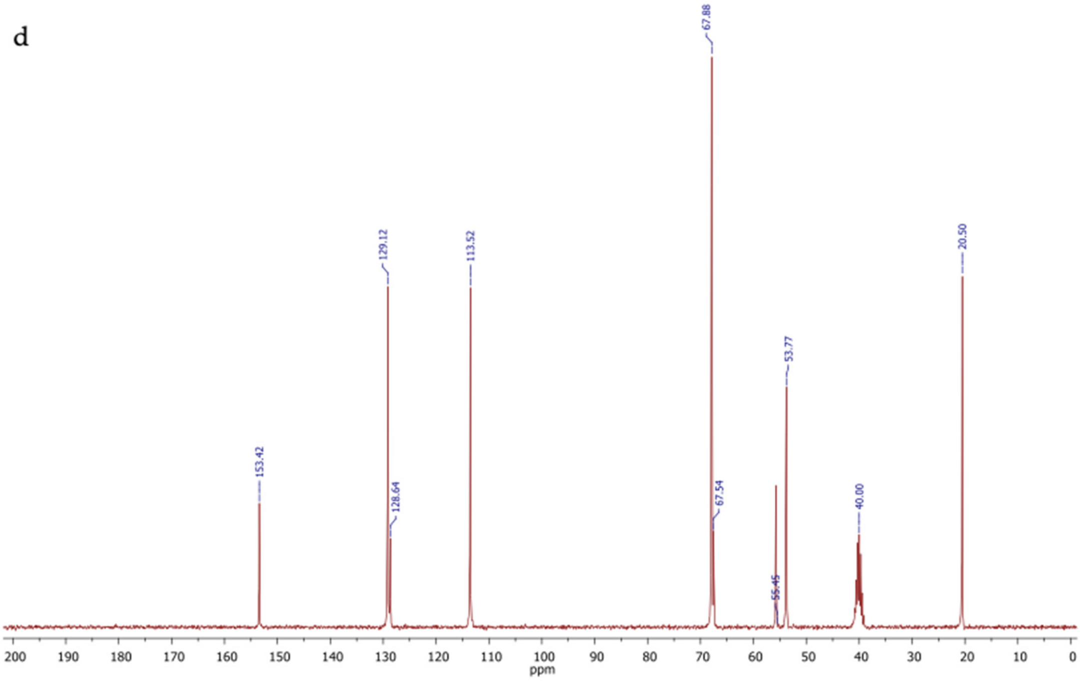

Figure S3. ^{13}C NMR spectra of choline chloride (**a**), dapsone (**b**), ChCl-PG DES (**c**) and DES-DAP ternary system (**d**).

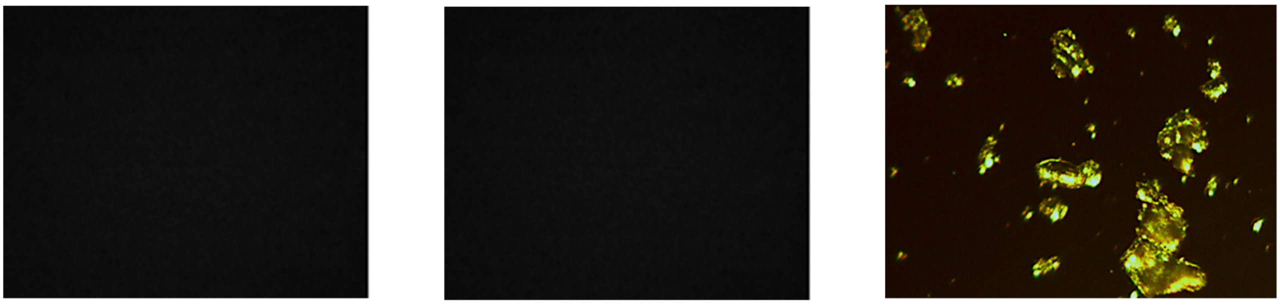

100 mg DAP + CC:AA DES

500mg DAP + CC:PG DES

500 mg DAP + PROPYLENE GLYCOL

Figure S4. POM images of (DRUG + PG) and (DRUG + DESs) at rt.

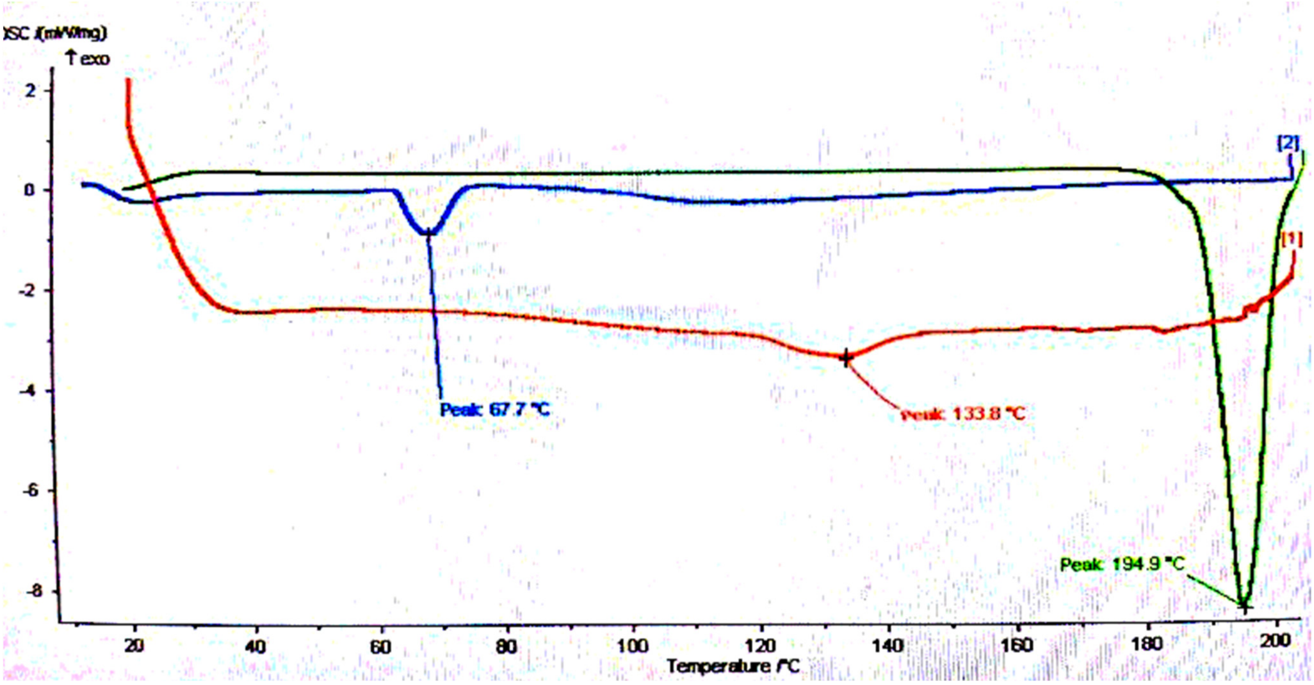

Figure S5. DSC thermograms of (1) CC:AA DES, (2) pure CC and (3) pure AA.
